# Low-Theta Electroencephalography Coherence Predicts Cigarette Craving in Nicotine Addiction

**DOI:** 10.3389/fpsyt.2019.00296

**Published:** 2019-05-03

**Authors:** Junjie Bu, Ru Ma, Chuan Fan, Shinan Sun, Yan Cheng, Yi Piao, Pengyu Zhang, Chialun Liu, Xiaochu Zhang

**Affiliations:** ^1^Hefei Medical Research Center on Alcohol Addiction, Anhui Mental Health Center, Hefei, China; ^2^Hefei National Laboratory for Physical Sciences at the Microscale and School of Life Sciences, University of Science & Technology of China, Hefei, China; ^3^School of Humanities & Social Science, University of Science & Technology of China, Hefei, China; ^4^Department of Medical Psychology, the First Affiliated Hospital of Anhui Medical University, Hefei, China; ^5^Academy of Psychology and Behavior, Tianjin Normal University, Tianjin, China

**Keywords:** electroencephalography coherence, nicotine addiction, smoking cue reactivity, external validation, functional connectivity

## Abstract

Addicts are often vulnerable to drug use in the presence of drug cues, which elicit significant drug cue reactivity. Mounting neuroimaging evidence suggests an association between functional magnetic resonance imaging connectivity networks and smoking cue reactivity; however, there is still little understanding of the electroencephalography (EEG) coherence basis of smoking cue reactivity. We therefore designed two independent experiments wherein nicotine-dependent smokers performed a smoking cue reactivity task during EEG recording. Experiment I showed that a low-theta EEG coherence network occurring 400–600 ms after onset during long-range (mainly between frontal and parieto-occipital) scalp regions, which was involved in smoking cue reactivity. Moreover, the average coherence of this network was significantly correlated with participants’ level of cigarette craving. In experiment II, we tested an independent group of smokers and demonstrated that the low-theta coherence network significantly predicted changes in individuals’ cigarette craving. Thus, the low-theta EEG coherence in smokers’ brains might be a biomarker of smoking cue reactivity and can predict addiction behavior.

## Introduction

Nicotine addiction is a psychiatric disorder that is one of the leading causes of avoidable morbidity and mortality globally (1). One common feature of nicotine addiction is smoking cue reactivity, which refers to how nicotine-dependent patients show significant physiological and subjective reactions to cigarette-related cues (2). According to addiction theory, smoking cue reactivity is a central characteristic of nicotine addiction (3), and emerging evidence suggests that it is a precipitating factor in many relapse episodes (4). The reverse may also be true: that is, brain reactivity to smoking cues might predict the ability to maintain nicotine abstinence (5). Many studies have since explored the brain basis of smoking cue reactivity, given the potential clinical benefit this knowledge would have for the treatment of nicotine addiction.

Functional magnetic resonance imaging (fMRI) studies have shown that smoking cue reactivity involves many brain regions, including the anterior cingulate cortex, the superior frontal gyrus, the posterior cingulate cortex, etc. This suggests that the brain connectivity network is an important basis of smoking cue reactivity (4, 6, 7). However, the low temporal resolution of blood-oxygen-level-dependent (BOLD) fMRI provides limited understanding of the temporal process of smoking cue reactivity (8). Numerous electroencephalography (EEG) findings suggest that smoking cue reactivity might be a relatively fast cognitive process (taking milliseconds). These findings showed that smoking cue reactivity tends to occur 300 to 800 ms after cue onset (9, 10). Although previous EEG studies have shown that smoking cue reactivity is related to the P300 (11), the slow positive wave (12), and the alpha power (10), these studies at best reveal that event-related potentials (ERPs) or time-frequency power were related to smoking cue reactivity, which provides rather limited understanding of the brain networks involved.

EEG coherence, as a measure of the brain network, which involves calculating the linear relationship between two electrode signals based on their cross-spectrum and estimating the synchronization between neural populations at a high temporal resolution, is believed to reflect functional cortical connectivity at the centimeter scale (13). This coherence is regarded as a direct reflection of the operation of corticocortical fiber systems or as an indirect reflection of the interactions of various brain networks including other cortical or subcortical structures (14). Therefore, EEG coherence has advantages on high temporal resolution and measuring brain network between populations of neurons. Recently Li et al. (15) used resting EEG coherence measure to explore the mechanism of hypnotic aversion suggestions on reducing cigarette craving. However, the EEG coherence basis of smoking cue reactivity is still unknown. In addition, so far few studies used other EEG measures of connectivity in nicotine addiction.

We designed two independent experiments to explore the EEG coherence basis of smoking cue reactivity. In experiment I, we found that smoking cue reactivity was related to increased low-theta (3–5 Hz) coherence at 400–600 ms after stimulus onset in long-range (between frontal and parieto-occipital) scalp regions. Additionally, craving—a core symptom of addiction that is often accompanied by drug cue reactivity—for cigarettes was significantly correlated with the average coherence of the low-theta network. In experiment II, an external validation demonstrated that the average coherence of the low-theta network predicted individuals’ cigarette craving.

## Methods

### Experiment I

#### Participants

Through online advertisements, we recruited 25 right-handed, unmedicated male nicotine-dependent smokers (≥10 cigarettes/day, ≥2 smoking years, aged 18–30 years) who met the *Diagnostic and Statistical Manual of Mental Disorders*, Fourth Edition, Text Revision (DSM-IV-TR) criteria for nicotine addiction as the smoker group, as well as 22 right-handed, healthy male adults (aged 18–30 years) as the nonsmoker group. All were studying at the University of Science and Technology of China. Because of the very low prevalence (only 2.7%) of female smokers in China, we enrolled only male smokers in this study. All had normal or corrected-to-normal vision. All participants gave their written informed consent before the experiment began and received financial compensation for completing it. The research protocol was approved by the Human Ethics Committee of the University of Science and Technology of China.

#### Experimental Procedure

Both groups completed a classical smoking cue reactivity task during EEG recording. Before the task, all participants recorded their demographic information including age and education years and completed the Toronto Alexithymia Scale (TAS) and the Positive and Negative Affect Schedule (PANAS). Furthermore, in the smoker group, we assessed cigarette craving before and after the smoking cue reactivity task using the Tobacco Craving Questionnaire (TCQ). Using the TCQ, we evaluated the change (i.e., increase) in cigarette craving pre-task to post-task. Participants were required to be abstinent from smoking cigarette for 2 h before the experiment. To ensure smokers’ 2 h abstinence before the experiment, an experimenter would tell the participants not to smoke by telephone. And these smokers were also required to self-report the time of last cigarette smoked after arriving at the lab.

**Figure 1:**
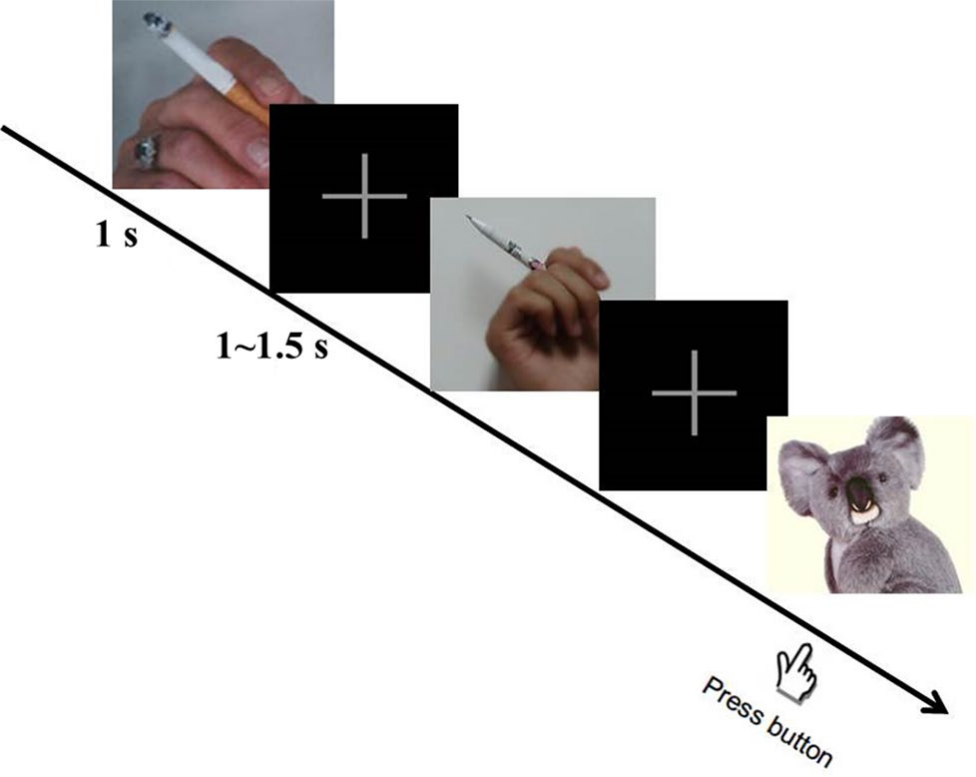
Smoking cue reactivity task. During the task, participants were required to press the space button as soon as possible when the animal picture appeared. Three hundred pictures in total (including 130 smoking, 130 neutral, and 40 animal pictures) were displayed randomly. After participants viewed 100 pictures, they rested for 1 min.

In the smoking cue reactivity task [adopted from our previous study (15); **Figure 1**], three kinds of cues [smoking (130 pictures), neutral (130 pictures), and animal (40 pictures)] were randomly presented to the participants. They were instructed to press the space bar on a keyboard as soon as possible when the animal picture appeared on the screen, in order to get them to focus their attention on the task. All these pictures were taken from our previous study (16). Each picture was displayed for 1 s and a fixation was presented during interstimulus intervals, which randomly varied from 1 to 1.5 s. For every 100 pictures displayed (about 3.7 min), participants were asked to rest for 1 min. The smoking cue reactivity task lasted for about 15 min in total. All participants pressed the button correctly when the animal picture appeared (100% accuracy).

#### Electroencephalography Acquisition

The experiment task was run using the Psychophysics Toolbox for Matlab (http://psychtoolbox.org/). The EEG data were recorded using a SynAmps RT amplifier (NeuroScan, Charlotte, NC, U.S). Sixty-four Ag/AgCl electrodes were placed on participants’ scalp at specific locations according to the extended International 10–20 System. Additionally, the electrical activities were recorded over the right and left mastoids. Vertical electrooculograms (EOGs) were recorded using bipolar channels placed above and below the left eye, and horizontal EOGs were recorded using bipolar channels placed lateral to the outer canthi of both eyes. The reference electrode was attached to the tip of each participant’s nose and the ground electrode was attached to AFz. Impedance between the reference electrode and any recording electrode was kept under 5 kΩ. All the signals were digitized at 500 Hz during data collection.

#### Data Analysis

The raw EEG data during the smoking cue reactivity task were pre-processed, first *via* visual inspection to remove obvious technical artifacts, after which a high-pass filter was used to remove low-frequency noise and an independent component analysis was used to correct for blink artifacts. The continuous EEG data were then extracted into epochs from −200 ms (pre-stimulus) to 1,000 ms (post-stimulus) and baseline corrected using the interval from −200 to 0 ms. Epochs containing amplitude changes exceeding ±100 μv were rejected.

For the remaining epochs, spectral coherence, which reflects the connectivity between two electrodes, was calculated for every condition (e.g., smoking condition and the neutral condition) during the smoking cue reactivity task. To reduce the volume conduction effect, the electrode pairs for calculating spectral coherence were separated by at least 10 cm (14). Given that every trial lasted for about 1 s and the possible electromyography (EMG) artifacts for high frequency data, we calculated the spectral coherence from 3 to 30 Hz. To calculate the spectral coherence between two electrodes across epochs at each time-frequency region (TFR), the average cross-spectrum was calculated from the complex conjugate of the wavelet coefficients, after which it was squared and normalized using the average residual power spectrum of the individual electrodes. For each participant, the spectral coherence value between two electrodes at each TFR was calculated using the EEGLAB function *newcrossf*. For each TFR, the coherence matrix was constructed by calculating the coherence value between each electrode pair (>10 cm). The long range is that the interelectrode distances are greater than 10 cm. Previous studies have suggested that the short distances (<10 cm) may influence coherence measurements such that increased coherence can be measured even when the underlying sources are uncorrelated (14). Therefore, the long range (>10 cm) seems to reflect genuine group differences in coherent neuronal activity. To identify the significant TFR of the coherence network, we followed previous coherence analysis methods (14, 15): that is, the overall pattern of spectral coherence was computed by averaging all electrode pairs and then compared between the two experimental conditions across participants using nonparametric permutation test and false discovery rate (FDR) correction. Furthermore, the topography of the difference between the two conditions was plotted based on the significant TFR. The correlation between the average coherence within the network and the change in cigarette craving was measured using Pearson’s correlation coefficient.

### Experiment II

We recruited an independent group of nicotine-dependent patients (13 males; mean ± SD age, 26.8 ± 2.8 years; mean ± SD years of education, 15.9 ± 1.5 years) for experiment II. The EEG data during the smoking cue reactivity task were taken from another neurofeedback study of ours, obtained in the same manner as in experiment I. The participants of this study also completed several other cognitive tasks while the EEG was recording; however, these task data were used only in the other study. We used the EEG coherence network for smoking cue reactivity obtained in experiment I to define that in experiment II. Subsequently, the average coherence of the network was used to predict the change in cigarette craving through the same correlation model as in experiment I. The change in cigarette craving predicted by the EEG coherence network was then entered into a Pearson’s correlation analysis with the observed change in cigarette craving. As in experiment I, we measured cigarette craving before and after the smoking cue reactivity task using the TCQ.

## Results

### Experiment I

As shown in **Table 1**, there were no significant differences between the smoker and nonsmoker groups in age, education years, and TAS and PANAS scores, suggesting that both groups were homogenous in their characteristics.

**Table 1:** Baseline demographic characteristics of the two groups.

Characteristic	Smoker group	Nonsmoker group	*p*
**Age (years)**	26.4 (3.2)	26.8 (1.8)	0.65
**Education (years)**	15.7 (1.7)	15.3 (1.1)	0.41
**Cigarettes/day**	15.4 (5.7)	0	0
**Years of cigarette use**	6.3 (2.5)	0	0
**TAS**	66.8 (9.6)	63.5 (8.5)	0.25
**PANAS**			
** Negative**	24.5 (6.9)	24.6 (8.2)	0.96
** Positive**	32.6 (5.7)	31.7 (6.0)	0.66

Values are means (1 standard deviation). TAS, Toronto Alexithymia Scale; PANAS, Positive and Negative Affect Schedule.

**Figure 2:**
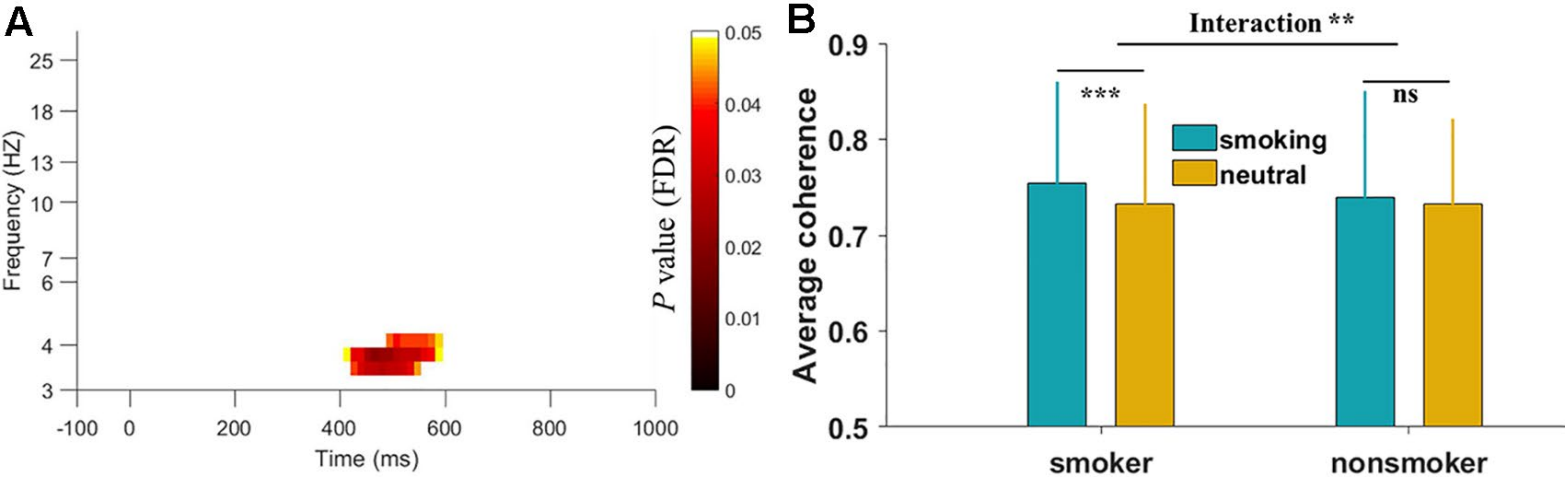
Time-frequency region for overall electroencephalography (EEG) coherence in the smoker group and interaction results between two groups. **(A)** The significant region indicated that the overall EEG coherence in the smoking condition was greater than that in the neutral condition during the smoking cue reactivity task. The *p* values were corrected with the false discovery rate. Log transformation was applied on the y-axis frequency. **(B)** The interaction results on the average EEG coherence of selected time-frequency region between two groups. **p < 0.01, ***p < 0.001, ns: not significant.

To identify the significant TFR for coherence during smoking cue reactivity, we compared the overall patterns between the smoking and neutral conditions by averaging all electrode pairs. As shown in **Figure 2**, a significant low-theta TFR pattern (occurring 400–600 ms after stimulus onset in the 3–5 Hz band) was found in the smoker group. Furthermore, the low-theta TFR pattern was greater in the smoking condition than in the neutral condition. However, we found no significant TFR pattern differences between the smoking and neutral conditions in the nonsmoker group. Then we performed a two-way mixed-design ANOVA analysis using group (smoking vs. nonsmoking people) as a between-subjects factor and cue type (smoking vs. neutral) as a within-subjects factor. We found there was a significant group-by-cue type interaction on the average EEG coherence [F(1,45) = 7.23, p < 0.01].

**Figure 3:**
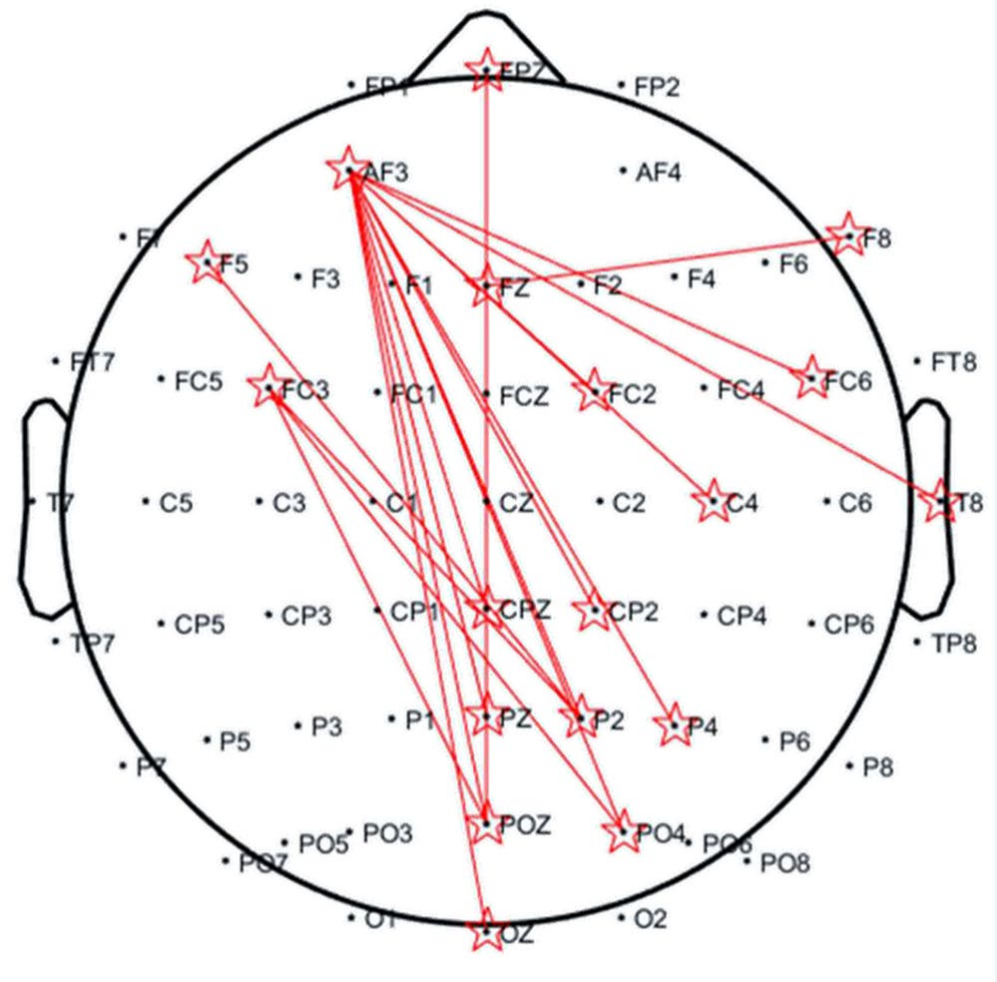
Topography of significantly increased coherences in the 3–5 Hz band occurring at 400–600 ms for the smoker group. The distance of each electrode pair for calculating the coherence (reflected by red lines) was >10 cm.

**Figure 3** shows the topography of the condition differences in the significant low-theta TFR pattern. The smoking condition showed higher coherence for long-range (e.g., frontal and parieto-occipital) scalp regions than did the neutral condition among smokers, but not among nonsmokers.

**Figure 4:**
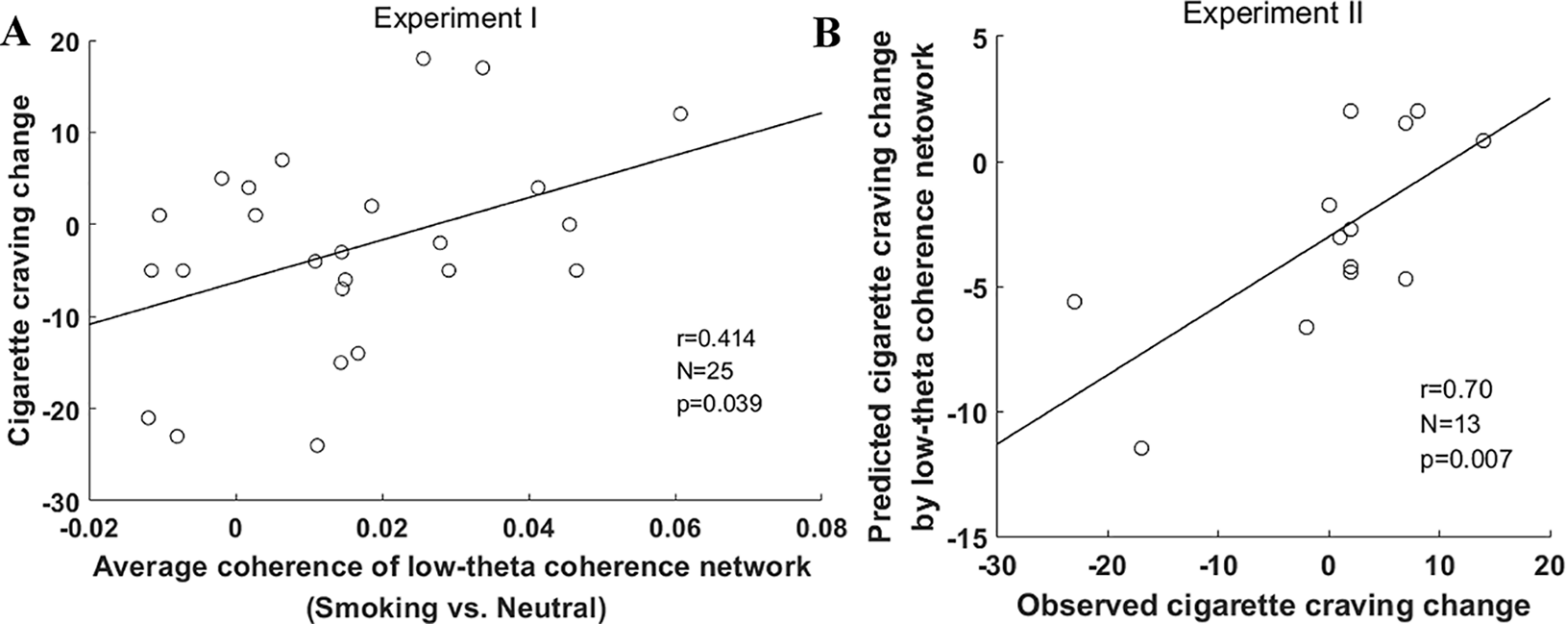
Prediction of change in cigarette craving change by low-theta coherence network in the two experiments. **(A)** The correlation between cigarette craving change and the average coherence of the low-theta coherence network in experiment I. **(B)** The correlation between observed and predicted change in cigarette craving in experiment II. The change in cigarette craving indicated an increase from pre-task to post-task (post–pre).

As for the relationship between the low-theta network coherence and cigarette craving, **Figure 4A** shows a significant positive correlation between the average coherence of the low-theta network and the change in cigarette craving (*r* = 0.41, *p* < 0.05).

### Experiment II

In experiment II, the change in cigarette craving ranged from −23 to 14 (mean ± SD, 0.23 ± 9.98), which was similar to experiment I (mean ± SD, −2.72 ± 10.93; *t* = −0.18, *p* = 0.42). In this external validation, the estimated correlation model (y = 229.58*x-6.26) used to predict the change in cigarette craving was derived from the entire sample in experiment I. We found that the predicted change in cigarette craving using the low-theta network coherence was significantly correlated with participants’ observed change in cigarette craving (*r* = 0.70, *p* = 0.007, **Figure 4B**); furthermore, they did not significantly differ (*t* = −1.07, *p* = 0.30). These external validation results indicate that the low-theta coherence basis of smoking cue reactivity significantly predicts the change in cigarette craving for a given participant based on the average coherence of the low-theta network.

## Discussion

In this study, we investigated the EEG coherence basis of smoking cue reactivity using the classical smoking cue reactivity task. First, we found increased coherence in the low-theta EEG network in the frontal-partial regions during smoking cue reactivity. Second, this low-theta coherence network was significantly associated with changes in cigarette craving. Finally, an external validation in an independent group of participants revealed that the average coherence of the low-theta network significantly predicted the change in cigarette craving.

In current study, nicotine-dependent individuals showed increased low-theta coherence during smoking cue reactivity when compared with nonsmokers. Our current findings build on these past studies by identifying some of the brain networks involved in smoking cue reactivity. Previous ERP and EEG oscillation studies revealed that smoking cue tend to elicit reactivity around the period between 300 and 800 ms after the cue appears (12), indicating that it is a relatively fast process. Our findings were well in line with the past studies, suggesting an early attentional deployment (arising between 400 and 600 ms reflected on low-theta band) on smoking cue. In addition, the frontal–parietal region connection (>10 cm) of the observed coherence network appears to be in line with the findings of a previous fMRI study showing that smoking cue reactivity was associated with the connectivity of the anterior cingulate cortices and precuneus (4). Moreover, the theta coherence from our second experiment has been shown to predict subjective craving level. Taken together, the current study may provide a novel and reliable biomarker for identifying smoking cue reactivity at both high temporal resolution and a certain degree of spatial resolution.

Smoking cue reactivity is accompanied by changes in cigarette craving (17). We found that the average coherence within the low-theta network was significantly correlated with changes in cigarette craving, thus supporting the idea the low-theta network is involved in nicotine addiction. Similarly, previous studies have shown that EEG theta coherence is associated with years of heroin use among people with heroin addiction (18). EEG theta coherence has also previously been found to be a biological marker of alcohol addiction (19). Collectively, these findings suggest the EEG theta coherence might play an important role in drug addiction.

Addiction studies have shown that cue reactivity involves numerous complex cognitive components (20), including attention, memory, emotion, etc. Long-range theta coherence has previously been found to be associated with working memory and sustained attention (21–23). Additionally, the scalp distribution of long-range theta coherence occurred primarily in regions where the memory and attention networks are localized (24). Based on these findings, we speculate that the low-theta coherence network we observed might represent memory and attention processes related to nicotine cue. However, to fully understand these mechanisms, concurrent EEG and fMRI experiments could be conducted to reveal precise network connectivity in future studies.

Recently, the replication of the results of neuroimaging studies has generated hot debate among researchers (25). Some studies are failing to be replicated, which might impede the healthy development of neuroimaging research as a whole (26). A potential reason for the lack of replication is that the generalizability of an internally validated prediction might be poor for a new sample (27). Therefore, we designed an independent experiment to externally validate the relationship between the low-theta coherence network and cigarette craving. The significant positive correlation observed between the observed change in cigarette craving and the predicted change based on the low-theta coherence network provide further evidence that this network might be a stable biomarker of nicotine addiction. This biomarker could therefore be an appropriate brain manipulation target for advanced neurofeedback or transcranial alternating current stimulation modulation technology for nicotine-dependent patients. However, these interventions require further investigation.

The present study is not without limitations. First, the sample size is not large, especially in the independent experiment. In addition, the participants’ age is only from 18 to 30. Further studies should increase the number of nicotine-dependent patients and explore the EEG coherence mechanism of nicotine addiction in the different age group. Second, the spatial resolution of EEG coherence network was on a centimeter scale. Further studies could consider having the concurrent EEG-fMRI or magnetoencephalography (MEG) experiments to reveal complete brain basis of smoking cue reactivity.

To the best of our knowledge, this study is the first one to assess cortical connectivity during smoking reactivity through applying EEG coherence methods. The low-theta coherence we identified was a stable and novel biomarker for smoking cue reactivity that could be targeted for treating nicotine addiction.

## Ethics Statement

The research protocol was approved by the Human Ethics Committee of the University of Science and Technology of China. All participants gave written informed consent prior to the study.

## Author Contributions

JJB and XCZ conceived and designed the study. JJB, RM, CF, SNS, YC, YP, and PYZ performed the research and analyzed the data. JJB, XCZ, RM, and CLL wrote the manuscript.

## Funding

This work was supported by grants from The National Key Basic Research Program (2016YFA0400900 and 2018YFC0831101), The National Natural Science Foundation of China (31471071, 31771221, 61773360, and 71874170), The Fundamental Research Funds for the Central Universities of China. A portion of the numerical calculations in this study were performed with the supercomputing system at the Supercomputing Centre of USTC.

## Conflict of Interest Statement

The authors declare that the research was conducted in the absence of any commercial or financial relationships that could be construed as a potential conflict of interest.
